# Fish Consumption and Sustainability: A Modeling Study Evaluating the Effects of Fish and Canned Fish Consumption on Nutritional Adequacy and Selected Metrics of Environmental Impact

**DOI:** 10.3390/nu18193182

**Published:** 2026-09-26

**Authors:** Ellis Bianchi, Massimiliano Tucci, Alberto Dolci, Cristian Del Bo’, Daniela Martini, Patrizia Riso

**Affiliations:** 1 Division of Human Nutrition, Department of Food, Environmental and Nutritional Sciences (DeFENS), Università Degli Studi di Milano, 20133 Milan, Italy; ellis.bianchi@unimi.it (E.B.); massimiliano.tucci@unimi.it (M.T.); cristian.delbo@unimi.it (C.D.B.); patrizia.riso@unimi.it (P.R.); 2 Bolton Food Research & Innovation Center, The Westgate, Singapore 608531, Singapore

**Keywords:** sustainable diets, aquatic food, fish consumption, canned tuna, dietary pattern, dietary modelling, nutritional adequacy, environmental impact

## Abstract

**Background/Objectives:** Although aquatic foods are recognized for their nutritional value and relatively low environmental impact, their role in healthy and sustainable dietary patterns remains poorly characterized, particularly regarding canned fish products. This study aimed to evaluate the impact of increasing fresh and/or canned fish consumption within two healthy and sustainable dietary patterns representative of the Italian context, one EAT-Lancet-based (MED_EAT-IT) and one based on Italian Food-Based Dietary Guidelines (FBDGs). **Methods:** Three alternative scenarios were generated for each baseline dietary pattern by increasing fish intake from three to four servings/week, replacing poultry, while varying fresh fish and canned tuna contributions. Nutrient intakes, nutritional adequacy, and environmental impact indicators were compared across scenarios. **Results:** Replacing poultry with fresh fish generally improved the nutrient profile of the MED_EAT-IT pattern, increasing EPA+DHA (+14%), vitamin B12 (+11%), and vitamin D (+12%) intakes while slightly reducing cholesterol. These improvements were less evident in the FBDGs pattern, likely due to its fixed portion-based structure. Compared with fresh fish, substituting canned tuna resulted in slightly lower intakes of selected micronutrients and cholesterol, while nutritional adequacy was maintained for most nutrients. Carbon and water footprints were largely unchanged, with modest reductions observed as fish intake increased, particularly when canned tuna was included (up to −6.2% CF and −5.5% WF). **Conclusions:** These findings suggest that increasing fresh and canned fish consumption within optimized dietary patterns may improve micronutrient intake while maintaining, or slightly reducing, environmental impact, supporting the promotion of aquatic foods, including shelf-stable products, in healthy and sustainable diets.

## 1. Introduction

Nutritional deficiencies and diet-related diseases remain major public health concerns, contributing to approximately 48.7 million disability-adjusted life years (DALYs) globally [1]. Although recent projections indicate gradual improvements in global nutritional status, food and diet remain major contributors to the global burden of disease [1]. Alongside these health challenges, growing concerns have been raised about the unsustainability of the global food system [2]. Indeed, food production currently represents a leading driver of climate change, resource impoverishing and biodiversity loss, creating an urgent need to reduce these side effects within safety margins that ensure its sustainability [3,4,5]. To achieve this aim, alongside interventions targeting other stages of the food system, research has increasingly focused on promoting healthy and sustainable dietary patterns at the household and individual levels, with the Mediterranean diet emerging as a key reference model due to its inherent characteristics, largely consistent with those of the Planetary Health Diet proposed by the EAT-Lancet Commission [5,6]. The latter was specifically designed to combine positive health outcomes with reduced environmental pressure through a predominantly plant-based dietary structure, and substantial overlap has been reported between the two dietary frameworks [5,7].

Despite the recognized environmental benefits of plant-based diets, a complete exclusion of animal-source foods (ASFs) may pose risks to nutritional adequacy, particularly for vulnerable populations [8]. The potential trade-offs between reducing environmental impact and ensuring adequate nutrient intake illustrate the complexity of defining what should qualify as a sustainable healthy diet [9,10]. In this context, the exploitation of aquatic ASFs (or “blue foods”) has received limited attention despite their theoretically favorable environmental and nutritional profile, and their historical integration into traditional European diets, spanning from the Mediterranean to Northern countries’ dietary patterns [11,12]. Indeed, they generally present a lower environmental impact than terrestrial ASFs (although this advantage can vary substantially depending on species, production or fishing method, and processing [11,13]), while providing a high density of essential nutrients that are often limited in diets with a low environmental impact [8,14,15]. In fact, beyond their established role as primary sources of long-chain omega-3 fatty acids (EPA and DHA), aquatic ASFs are also a source of important micronutrients such as vitamin B12, vitamin D, iodine, as well as highly bioavailable iron and zinc, which could help to address multiple dimensions of global micronutrient deficiencies, especially in low-income countries [13,14,16]. At the same time, in high-income countries, the replacement of high-impact terrestrial meats (e.g., beef) with fish and seafood could represent a potential initial step to concretely move towards more sustainable diets [11,17]. Despite these theoretical benefits, existing research has primarily focused on aquatic animal-source foods as individual food items or in comparison with other food groups. However, quantitative evidence on the effects of increasing fish consumption within the context of a whole healthy and sustainable dietary pattern remains limited.

Although global average consumption of aquatic ASFs has increased, intake levels vary largely across regions and population groups, causing some people to miss out on the benefits of eating fish [18,19]. A similar trend is observed at the Italian national level, where population intake often fails to meet the recommended frequency of consumption, particularly among younger age groups [20]. One of the reasons that negatively affect the use of such foods is probably linked to the high cost and perishability of fresh products [19,21]. Within this framework, canned fish products have attracted debate, being often regarded unfavorably because of their nutritional characteristics (e.g., higher fat and sodium content compared to fresh fish) and the perception that increased processing may translate into greater environmental impact and pollution [22]. However, shelf-stable options like canned fish may play an important role in improving the affordability and accessibility of this food category for a broader population [23,24]. Clarifying these aspects is crucial to guide both consumers and policymakers toward informed and sustainable dietary choices [25]. However, available evidence has not yet evaluated whether and to what extent canned fish can be effectively incorporated into a healthy and sustainable dietary pattern.

In light of these considerations, this study aimed to evaluate, through dietary modeling, whether increasing the consumption of fresh and/or canned fish within the context of whole healthy and sustainable dietary patterns could improve nutritional adequacy without increasing environmental impact. Specifically, we assessed the effects of progressively replacing poultry with fresh fish and canned tuna on nutrient intake, nutritional adequacy, and environmental sustainability, using carbon and water footprints as indicators, within two dietary patterns representative of the Italian context: the MED_EAT-IT dietary pattern, adapted from the EAT-Lancet Commission recommendations, and a dietary pattern based on the Italian Food-Based Dietary Guidelines (FBDGs).

The MED_EAT-IT dietary pattern represents a feasible adaptation of the EAT-Lancet Commission recommendations to the Italian context, translating the Planetary Health Diet into a structured and scalable framework [26]. The present study thus further expands its application by evaluating whether increasing fish and seafood consumption can improve selected aspects of nutritional adequacy while maintaining the environmental sustainability of the overall dietary pattern.

## 2. Materials and Methods

### 2.1. Development of Dietary Scenarios

To set the different consumption scenarios, we used a previously developed calculation tool designed to support the construction of feasible, real-life dietary patterns based on the EAT-Lancet Commission framework, described in detail elsewhere [26]. Briefly, this Excel-based tool operates by integrating a structured food ontology with food composition and environmental impact databases. The main function of the tool is to automatically compute the nutrient profile of a given dietary pattern considering a wide range of macro- and micronutrients, as well as the diet-related environmental impact in terms of carbon and water footprints. These outputs are calculated on the basis of the portion sizes and frequencies of consumption defined for the different food categories. In addition to quantifying nutritional and environmental characteristics, the tool enables the user to select specific food items within each category and to modify nutrient composition or environmental impact values when needed. These functionalities allow for the rapid development and evaluation of multiple dietary patterns and alternative scenarios. The tool also includes functions to assess the overall nutritional adequacy of each scenario by comparing the resulting nutrient intakes with the Dietary Reference Values (DRVs) issued by the European Food Safety Authority and by the Italian Society of Human Nutrition for healthy adults aged 18–64 years [27,28]. A complete list of considered DRVs is available in Supplementary Table S1.

Nutrient composition data for food items were derived from the Food Composition Database for Epidemiological Studies in Italy, which was selected to provide comprehensive and context-specific macro- and micronutrient profiles for foods commonly consumed by the Italian population [29]. Environmental impact data (carbon and water footprints) were obtained from the SU-EATABLE LIFE database [30]. For each food item, the environmental database provides a distribution of impact values reflecting the variability associated with different production systems and supply chains. Environmental impacts of the dietary scenarios are primarily estimated using the median value for each food item. To account for uncertainty and heterogeneity in production-related impacts, additional calculations are also performed using other points of the impact distribution (minimum, first quartile, third quartile, and maximum values).

### 2.2. Reference Pattern (Baseline Scenarios)

To assess the effects on nutrient profile and on the included metrics of environmental footprint associated with increased consumption of fish and canned fish within a healthy and sustainable dietary pattern, we used as baseline scenarios both the MED_EAT-IT dietary pattern and the Italian FBDGs as reference. The MED_EAT-IT dietary pattern was developed based on the framework of the Planetary Health Diet proposed by the EAT-Lancet Commission in 2019 and represents a healthy and sustainable dietary pattern developed for the Italian population. Its characteristics have been described in detail elsewhere [26]. However, a summary of the frequencies of consumption and portion sizes is provided in the Supplementary Table S2.

With regard to fish and seafood, the baseline MED_EAT-IT dietary pattern included three portions per week. To account for the diversity of products within this category and to develop more realistic scenarios, the food products included in the modeling were selected based on an analysis of current Italian dietary habits. For canned fish, only canned tuna (canned in oil, drained) was included, in accordance with the Comprehensive European Food Consumption Database of the European Food Safety Authority (EFSA), showing that it is by far the most consumed type of canned fish. Indeed, in the ‘All Subjects’ analysis, canned tuna emerged as the predominant product within the category, corresponding to 94.4% of total canned fish consumed by the Italian population (see Supplementary Table S10). For fresh fish (including mollusks and crustaceans) consumption, data were more evenly distributed across species in the EFSA’s Comprehensive European Food Consumption Database. Therefore, the 13 most frequently consumed products were included in the analysis to ensure adequate representation of this food category (i.e., reported in order of consumption from the most to the least consumed: cod, salmon, swordfish, squid, sea bass, gilthead seabream, shrimps, octopus, tuna, sole, mackerel, cuttlefish, clams) [31]. Additional data regarding consumption data underpinning this selection are reported in the Supplementary Table S11.

Three different energy targets for the MED_EAT-IT dietary pattern were considered, namely 2500, 2000, and 1600 kcal (i.e., 10.5, 8.4, and 6.7 MJ). These targets were selected, consistently with the original MED_EAT-IT elaboration, to reflect different segments of the Italian population: 2500 kcal is in line with the energy needs of individuals with an active lifestyle, 2000 kcal is suitable for sedentary and moderately active subjects, who represent more than 50% of the Italian population, while 1600 kcal represents a plausible energy-restricted intake for individuals with overweight, which account for approximately 36% of the Italian adult population [26]. The consumption frequencies for the “seafood” category remain the same across all energy targets, allowing the development of comparable scenarios.

Finally, in addition to the scenarios based on the MED_EAT-IT dietary pattern, we also used as a baseline a dietary pattern aligned with the reference portion sizes and consumption frequencies provided by the Italian FBDGs [32]. The Italian FBDGs provide recommendations for three energy targets (i.e., 2500, 2000, and 1500 kcal, or 10.5, 8.4, and 6.3 MJ), each with different fish consumption frequencies. For this analysis, we considered only the intermediate energy target, as it corresponds to the same fish consumption frequencies as the MED_EAT-IT dietary pattern. This approach allowed us to construct equivalent scenarios with identical quantities of fresh and canned fish within both the FBDGs and MED_EAT-IT frameworks.

### 2.3. Characteristics of Increased Fish Consumption Scenarios

Three different scenarios, involving a progressive increase in the consumption frequency of fish and/or the inclusion of canned fish (with a fixed serving size of 50 g of drained product, in accordance with the portion size recommended by the Italian FBDGs), were developed for each reference pattern. To account for the structural differences between the two baseline models, the energy adjustment was managed using two distinct approaches. In the MED_EAT-IT scenarios, the fish portion sizes were slightly adjusted to strictly maintain the predefined energy targets (i.e., 2500, 2000, and 1600 kcal), as this framework is designed to balance food category distributions to meet specific energy targets. Conversely, the Italian FBDGs rely on standardized reference portion sizes and consumption frequencies intended for practical dietary guidance (see Supplementary Table S3 for frequency of consumption and portion size of FBDGs-based dietary pattern). Therefore, in the FBDGs-based scenarios, the standard fish portion sizes were kept fixed to preserve the nature of the guidelines, resulting in a negligible variation in total daily energy intake (<10 kcal) across the modeled scenarios.

The characteristics of the developed scenarios are summarized in Table 1. Regarding fish consumption, both Baseline and Scenario 1 included a total of three portions of fresh and/or canned fish, whereas Scenarios 2 and 3 increased the total to four servings by introducing one additional fish serving. To maintain the overall dietary balance, the additional fish serving replaced one weekly serving of poultry. Poultry was selected as the substitution target to provide a conservative estimate of the environmental trade-offs. Indeed, poultry represents the terrestrial ASFs with the lowest carbon footprint, thus avoiding an overestimation of the environmental benefits that would occur through red meat replacement. Likewise, since wild-caught fish and seafood are not associated with aquaculture-related water-use impacts, all fish and seafood products included in the analysis were assumed to be farmed to provide a conservative estimate of the water footprint.

### 2.4. Statistical Analysis

All scenarios were compared descriptively in terms of nutrient composition and environmental impact metrics. No formal statistical tests were performed, as the analyses were based on predefined dietary scenarios. Values are presented to allow direct comparison of the effects of increasing fish and canned fish consumption within the different reference dietary patterns.

## 3. Results

### 3.1. Nutritional Profile Across Different Scenarios

Table 2 presents the selected key nutrients variations across the modeled scenarios. The complete nutritional profiles for all scenarios are provided in Supplementary Tables S4–S7; however, changes in other nutritional parameters were negligible.

In general, we observed that within the considered modeled scenarios, the replacement of one portion of fresh fish with canned tuna results in a modest reduction in most micronutrients and cholesterol in both dietary patterns. Conversely, substituting one portion of poultry with a portion of fresh fish leads to an increase in almost all key nutrients considered in both dietary patterns, alongside a marginal reduction in cholesterol in the MED_EAT-IT dietary pattern and a marginal increase in the FBDGs one.

Figure 1 shows additional representation, highlighting the percentage deviations from baseline. Green gradients indicate increases in nutrients associated with beneficial effects or decreases in critical nutrients (i.e., sodium and cholesterol), whereas red gradients indicate decreases in nutrients associated with beneficial effects or increases in critical nutrients (i.e., sodium and cholesterol). Data indicate that relative changes are largely consistent across the different energy levels of the MED_EAT-IT dietary pattern. In contrast, the impact of the substitutions differs markedly between the MED_EAT-IT pattern and the one based on the Italian FBDGs. In the MED_EAT-IT pattern, Scenario 2 appears to be associated with a better nutritional profile, mainly due to increases in EPA and DHA and vitamin B12, while other nutrients remain largely stable, aside from a minor reduction in zinc. Conversely, in the pattern based on the Italian FBDGs, any substitution leads to a generalized, albeit modest, decline in nutrient intake of the nutrients considered.

### 3.2. Nutritional Adequacy

Figure 2 presents an analysis of the theoretical nutritional adequacy of the selected nutrients in the modeled dietary patterns, assessed in relation to the DRVs for the adult population, considering the Population Reference Intake where possible or, where unavailable, the Adequate Intake or the Suggested Dietary Target [28]. Across all scenarios and energy thresholds, nutrient content reached the DRVs, with the exception of specific gender requirements for iron and zinc, and vitamin D (Figure 2). Specifically, female iron and male zinc requirements were satisfied exclusively at the highest energy target (2500 kcal), whereas vitamin D remained below the recommended levels across all scenarios and energy targets.

### 3.3. Environmental Impact: Carbon and Water Footprint

Figure 3 illustrates CF and WF across the modeled scenarios. Overall, the environmental impact changed only slightly, with each scenario showing modest reductions compared with its respective baseline at the same energy target. Reductions in CF (panels A1–A4) were minimal in Scenario 2 for the 2500 and 2000 kcal MED_EAT-IT dietary pattern (0.0 kg CO_2_ eq./day) and highest in Scenario 3 for the FDBGs (0.18 kg CO_2_ eq./day). For WF (panels B1–B4), the smallest reduction was found in Scenario 1 and 3 for the 2500 and 2000 kcal MED_EAT-IT dietary pattern (44 L/day) and the highest in Scenario 3 for the FDBGs (145 L/day).

More specifically, while substituting a portion of poultry with one of fresh fish (Scenario 2) did not result in relevant reduction in CF (from 0.00 to 0.06 kg CO_2_ eq./day depending on the dietary pattern), the substitution of a portion of fresh fish with canned tuna (Scenarios 1 and 3) resulted in more pronounced reductions, ranging from 0.08 to 0.13 kg CO_2_ eq./day in Scenario 1, and from 0.08 to 0.18 kg CO_2_ eq./day in Scenario 3.

The WF also decreased across the various scenarios, but showed a different pattern. In this case, the reduction became progressively larger from Scenario 1 to Scenario 3, for every energy target and dietary pattern. These reductions ranged from 44 to 64 L/day in Scenario 1, from 81 to 92 L/day in Scenario 2, and from 136 to 145 L/day in Scenario 3.

Comprehensive numerical data of the scenarios, including median, Q1, Q3, min and max, are reported in Supplementary Table S8. Absolute and relative differences compared to baseline are reported in Supplementary Table S9.

## 4. Discussion

The present study provides further evidence and novel insights concerning the role of “blue foods” within healthy and sustainable diets. Specifically, we evaluated nutritional and environmental trade-offs within the complexity of two whole healthy and sustainable dietary frameworks (MED_EAT-IT and a pattern based on national FBDGs), complementing existing single-product studies with a dietary-pattern perspective [13,33]. Overall, this study shows that including higher amounts of seafood, considering both fresh and canned options, represents a viable strategy to maintain, or even enhance, both micronutrients intake and environmental impact. Although these baseline patterns are already optimized to balance human and planetary health, their predominantly plant-based structure may limit the intake of key micronutrients. Therefore, our hypothesis was that strategically increasing the consumption of aquatic foods, including affordable, shelf-stable options, could serve as an effective nutritional strategy to support the intake of these critical nutrients without exceeding the established environmental boundaries.

Reducing animal source foods is widely recognized as an effective strategy to lower diet-related environmental impact [5,7]. However, animal-source foods, and fish in particular, can effectively support the attainment of nutritional needs for the general population, serving as a source of highly bioavailable nutrients [14]. In this regard, once a safe operating space has been identified, the consumption of nutrient-dense animal-source foods may help address “hidden hunger”, including among population subgroups with specific nutritional needs (e.g., children, pregnant women, older adults) [34,35,36,37]. Our data partially support these positions, as they indicate that increasing fresh fish consumption led to a simultaneous increase in critical nutrients such as EPA + DHA, vitamin B12, and vitamin D. Furthermore, the MED_EAT-IT-based scenarios showed that this increase in fish consumption concurrently led to lower dietary cholesterol levels. This outcome could be particularly relevant for specific population groups, such as older adults, who can benefit from dietary patterns aimed at managing cardiovascular risk factors. In contrast, in the Italian FBDGs-based scenarios, increasing fish consumption produces the same effect because of differences in the modeling approach. Indeed, FBDGs rely on standardized portion sizes rather than the energy-balanced substitutions [32]. Therefore, replacing poultry with fish without adjusting portion sizes on an energy basis resulted in negligible reductions in nutrient intake, consistent with the nutrient density of the substituted foods.

Although a reduction in sodium intake would have been a desirable outcome, sodium values remained relatively stable across the scenarios analyzed. Nevertheless, the highest estimated intake, approximately 1.8 g/day of sodium (corresponding to about 4.5 g/day of salt), is consistent with international health recommendations and considerably lower than current consumption trends [38]. In contrast, EPA + DHA intake exhibited the most substantial variation compared to baseline. Specifically, the largest increase was observed in the MED_EAT-IT-based scenarios when substituting one portion of poultry with fresh fish. Although nutritional targets were met in all scenarios, the current literature highlights that increasing EPA and DHA intake can yield further health advantages beyond deficiency prevention, further decreasing the risk of cardiovascular events and other clinical outcomes [39,40,41,42].

Focusing on theoretical nutritional adequacy (i.e., comparison of estimated nutritional profile against the DRVs), our findings showed a consistent pattern across all scenarios and energy targets when compared to the baseline profile. Nutrients that were adequate at baseline remained within the recommended intake ranges, whereas pre-existing inadequacies persisted. Specifically, recommended intakes were achieved for all key nutrients considered, except for vitamin D and gender-specific variations for zinc and iron. Indeed, male zinc and female iron requirements were only marginally satisfied in the highest energy MED_EAT-IT scenario. Conversely, Vitamin D intake remained well below the recommended levels across all modeled dietary patterns. This finding was expected, as achieving the DRVs for vitamin D through diet alone is challenging because natural food sources are limited and generally provide relatively small amounts of this micronutrient [43]. Most of vitamin D requirements are met through endogenous synthesis in the skin following exposure to ultraviolet-B (UV-B) radiation, whereas dietary intake contributes only a limited proportion to overall vitamin D status [28,43,44,45]. In this context, it is worth noting that calcium intake remained adequate and highly stable across all modeled scenarios, showing only negligible variations compared to the baseline.

Although the environmental impact of seafood exhibits substantial heterogeneity mainly based on production method and species, “blue foods” are generally characterized by a lower environmental footprint when compared to terrestrial ASFs, particularly red meat [11,13,25,33]. Consistently with environmental impact data, we observed a marginal reduction in environmental impact, particularly regarding the water footprint in scenarios involving additional fresh and canned fish portions. A similar trend was observed for the carbon footprint, which exhibited a more pronounced decrease specifically when canned fish was included. The results obtained are consistent with recent analyses based on primary data that assessed the environmental impacts of the ten most consumed seafood products in the United States along the entire supply chain, including transportation, processing, retail, and home preparation [21]. This study reported that canned products (with tuna accounting for 97% of this category) exhibit lower environmental impacts than fresh and frozen fish in terms of energy use, CF, and WF. The authors attributed these findings to the fact that canned products are shelf-stable at ambient temperature and are less affected by issues related to food loss and waste [21]. Consequently, the scenarios illustrated in the present analysis reflect these aspects, as they focus on CF and WF as environmental impact indicators. However, the lower environmental impacts observed are also likely attributable to the smaller serving size of preserved fish considered in the analysis. Although the reductions observed in CF and WF in our analysis were modest, it is important to note that the modeling framework did not consider the replacement of ruminant meat with seafood. Instead, a conservative comparator was adopted by using poultry, which represents the terrestrial meat category with the lowest carbon footprint [46]. Furthermore, it should be acknowledged that, to ensure a conservative assessment, all fish and seafood products were assumed to originate from aquaculture, thereby yielding higher water footprint estimates. However, several species considered are likely to be sourced predominantly from wild-capture fisheries. As wild-caught products are associated with lower or negligible water footprint impacts compared with farmed counterparts, the reduction in water footprint associated with the proposed dietary shift would be even greater than estimated [47].

The findings of this study should be interpreted considering certain limitations. As a modeling study, our analysis is inherently constrained by the parameters and assumptions embedded in the simulation and cannot be extended beyond them. Moreover, aggregated data were utilized for environmental metrics, and consequently they may not fully capture the specific variability associated with different fishing gears, catch locations, or aquaculture practices [30,33]. Regarding canned fish, our conclusions are based on fixed portion sizes representative of the most commonly consumed products, according to available consumption data (i.e., canned tuna). Alternative assumptions, such as variations in the canned fish species considered (e.g., yellowfin tuna or skipjack), differences in portion sizes, or specific canning and processing methods, could yield different nutritional and environmental trade-offs, potentially altering the magnitude of the observed benefits [48]. In this regard, another limitation is represented by the absence of a formal sensitivity analysis. Furthermore, the current modeling framework lacks the quantitative data necessary to assess potential chemical or biological hazards, such as exposure to heavy metals from packaging materials, foodborne pathogens, or the use of specific additives and colorants.

Concerning environmental parameters considered in the present modeled analysis, this study focused on GHG emissions and water use as primary sustainability metrics. However, it is important to consider that a holistic assessment of seafood sustainability must also consider other parameters, including stock status and fishery management (e.g., MSC certification), particularly for wild-caught species prone to overfishing, as well as plastic pollution, seafloor impact, antibiotic resistance, etc. Nevertheless, it is important to note that where fisheries are effectively managed, evidence suggests a decoupling of seafood production from resource depletion, demonstrating that higher levels of production can be achieved while maintaining or even improving fish stock status [49]. Currently, data indicates that the majority of global landings come from biologically sustainable stocks (i.e., fishing pressure allow to maintain an adequate fish stock, avoiding depletion) [19].

Another aspect not considered by this modeling study regards a common concern regarding increased seafood consumption, particularly predatory species like tuna, which is the exposure to environmental contaminants such as methylmercury (MeHg). However, quantitative risk–benefit assessments, together with multiple systematic reviews and meta-analyses, consistently show that for the general population, the cardiovascular and neurodevelopmental benefits provided by polyunsaturated fatty acids and a wide range of key micronutrients clearly outweigh any potential adverse effects of contaminant exposure, provided that consumption stays within recommended frequencies (3–4 servings/week) and includes a variety of species [14,50,51,52,53,54]. Additionally, distinct differences exist between fresh and canned products regarding species size. Canned tuna is predominantly sourced from smaller, short-lived species or younger specimens of larger species, which naturally accumulate significantly lower levels of contaminants compared to the large, long-lived predatory fish often consumed as fresh steaks [51,55]. In fact, data from EFSA indicate that the higher amount of this metal is found in fresh swordfish, rather than canned tuna [56].

## 5. Conclusions

The findings of this study contribute to a more nuanced understanding of the role that aquatic foods play within healthy and sustainable dietary patterns. By modeling the integration of fresh and canned fish into the two dietary patterns considered, we noticed that increasing seafood consumption effectively supports the intake of critical micronutrients when substituting a portion of poultry with fresh fish, which was found to be more nutrient-dense than canned tuna, especially in terms of EPA and DHA. However, despite the nutritional variations observed between fresh and canned fish, the overall nutritional profile of the patterns generally remained within the bounds of nutritional adequacy. This finding is of practical relevance considering the relatively high consumption of canned tuna at the national level. Considering the specific metrics evaluated, canned tuna performed marginally better than fresh fish, reinforcing the role of both fresh and preserved fish as a practical dietary choice characterized by a relatively low climate and water impact. In fact, aquatic foods can effectively decouple high nutrient density from the environmental costs typically associated with animal-source foods. Considering aquatic foods within these modeled dietary patterns may help incorporate their combination of high nutrient density and comparatively low environmental impacts. While these findings are inherently model-based and scenario-dependent, they provide useful insights into the potential effects of increasing aquatic food consumption and may help inform the development of strategies aimed at balancing nutritional adequacy with environmental impact boundaries.

## Figures and Tables

**Figure 1:**
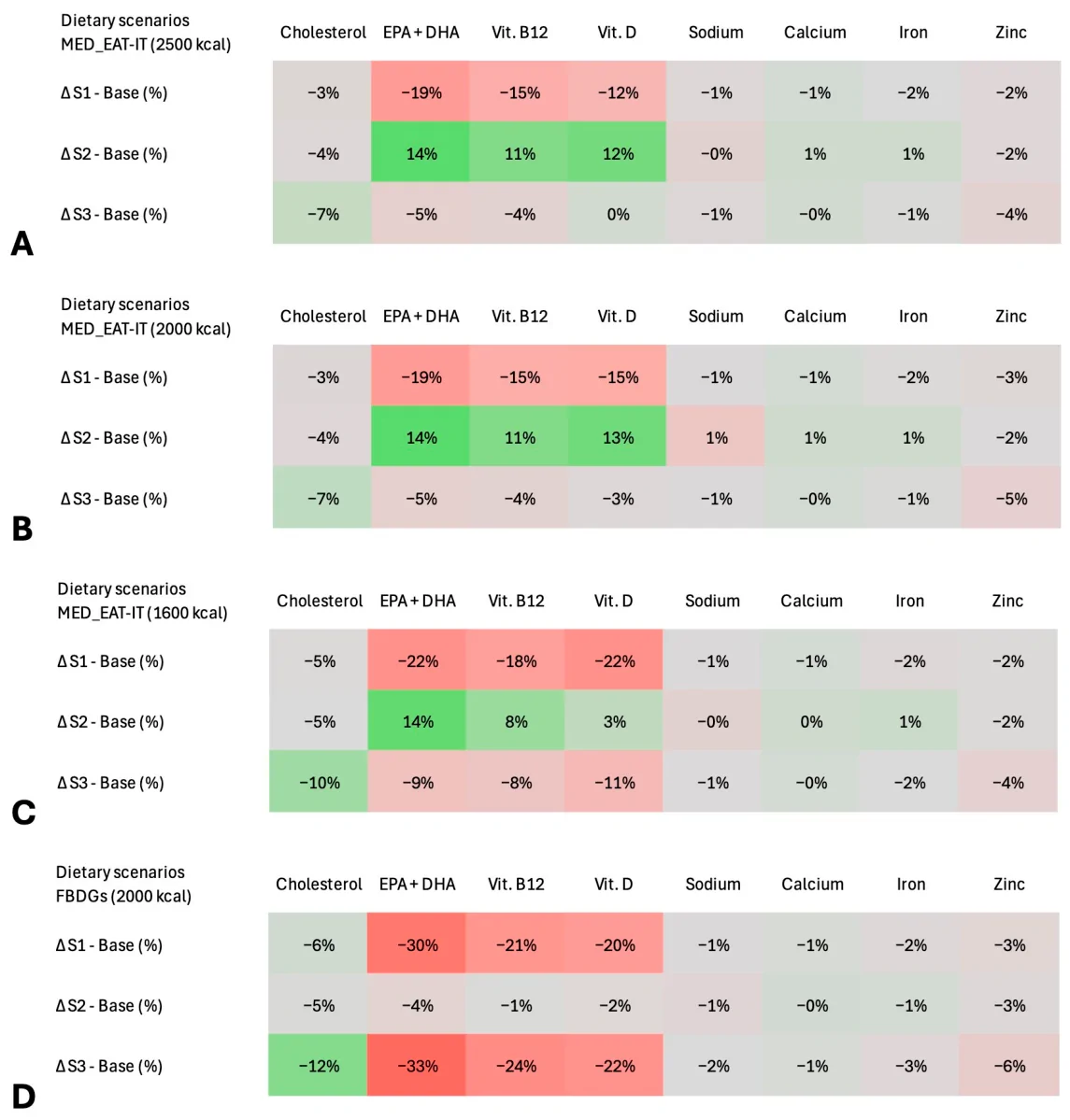
Heatmaps showing percentage differences (**Δ**) in terms of key nutrients between the modeled scenarios and the corresponding baseline scenarios, considering both MED_EAT-IT and FBDGs. For all nutrients, except for sodium and cholesterol, which follow the opposite gradient, green indicates an increase in the target nutrient, whereas red indicates a decrease. (**A**): 2500 kcal dietary scenario based on MED_EAT-IT dietary pattern; (**B**): 2000 kcal dietary scenario based on MED_EAT-IT dietary pattern; (**C**): 1600 kcal dietary scenario based on MED_EAT-IT dietary pattern; (**D**): 2000 kcal dietary scenario based on FBDGs dietary pattern.

**Figure 2:**
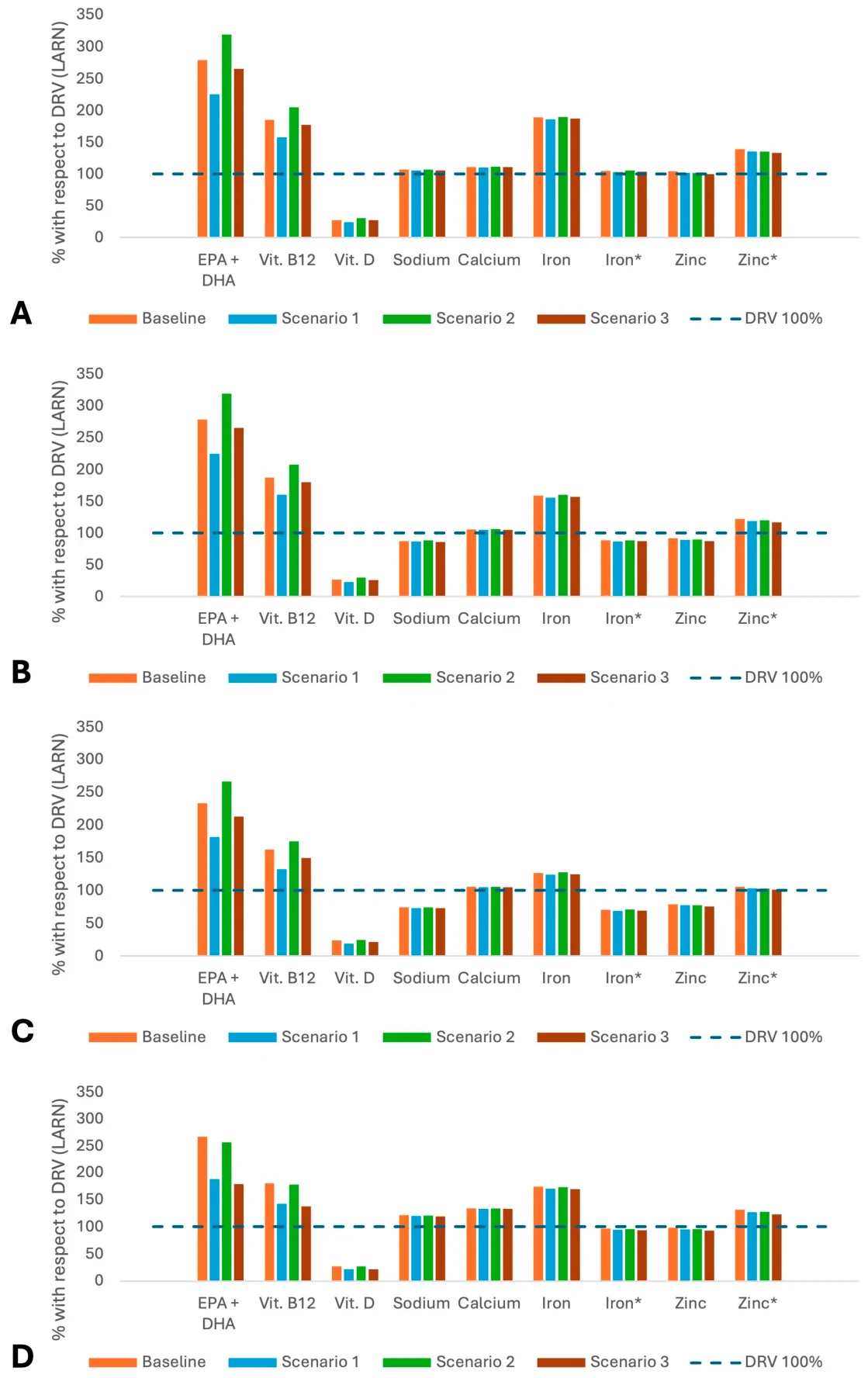
Bar graph showing percentage differences with respect to the DRV for the Italian population [28], considering the different scenarios, 2500/2000/1600 kcal as energy targets, and both MED_EAT_IT and FBDG-based scenarios. For iron and zinc, labels without an asterisk refer to the DRVs for adult males, whereas labels with an asterisk (*) denote the DRVs for adult females. (**A**): 2500 kcal dietary scenario based on MED_EAT-IT dietary pattern; (**B**): 2000 kcal dietary scenario based on MED_EAT-IT dietary pattern; (**C**): 1600 kcal dietary scenario based on MED_EAT-IT dietary pattern; (**D**): 2000 kcal dietary scenario based on FBDGs dietary pattern.

**Figure 3:**
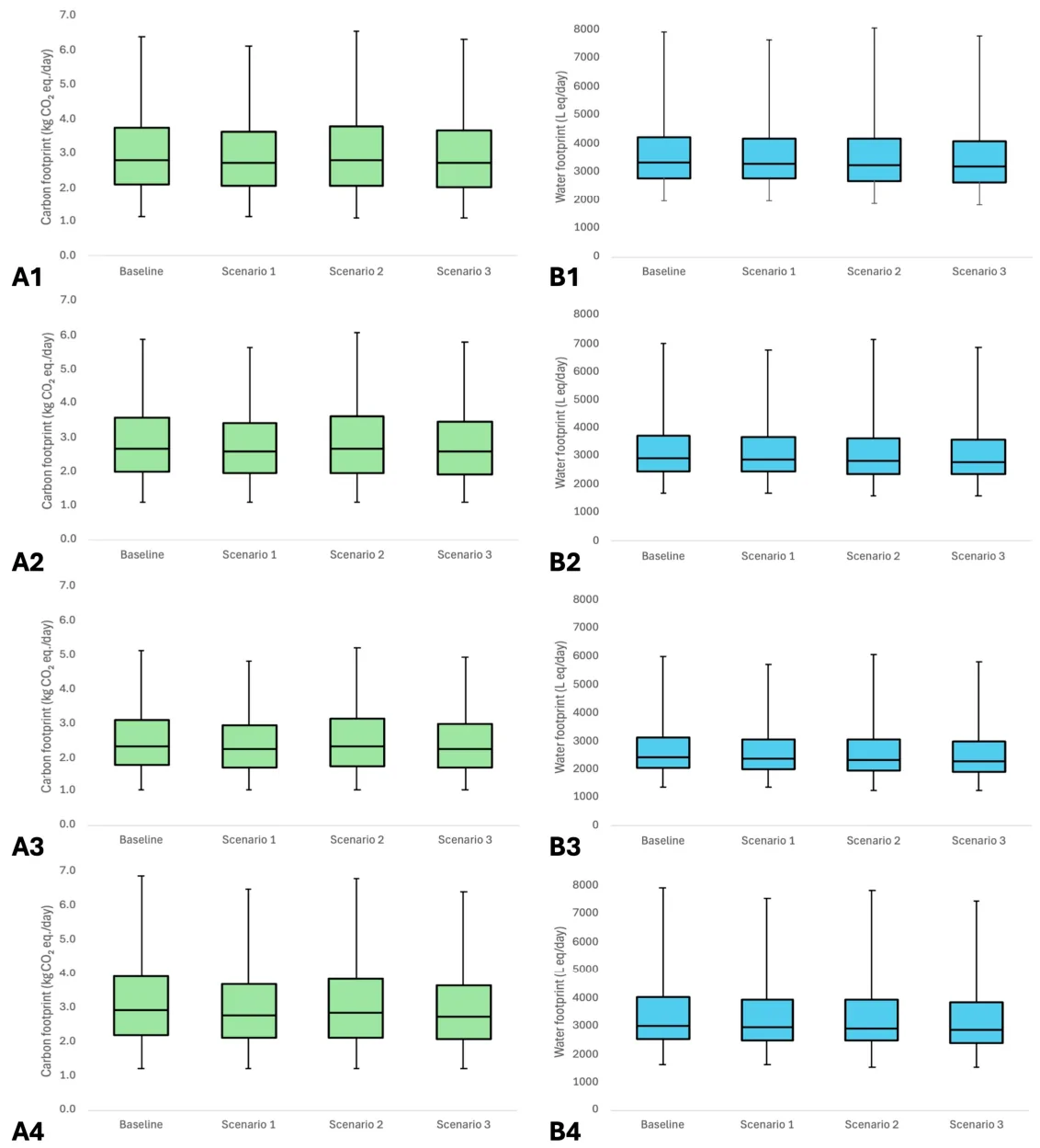
Boxplots illustrating the estimated carbon and water footprints across scenarios. Variability reflects the use of different food-specific environmental impact values reported in the database (minimum, Q1, median, Q3, and maximum), while diet composition was held constant. Left panels (**A1**–**A4**) display the Carbon Footprint, while right panels (**B1**–**B4**) show the Water Footprint. The rows correspond to the specific reference patterns and energy targets: (1) MED_EAT-IT at 2500 kcal; (2) MED_EAT-IT at 2000 kcal; (3) MED_EAT-IT at 1600 kcal; and (4) Italian FBDGs at 2000 kcal.

**Table 1:** Characteristics of the different scenarios created, in terms of portions of fish per week.

Scenario	N of Portions per Week (Total)
Baseline	3 Fresh fish + 0 canned tuna (3 in total)
Scenario 1	2 Fresh fish + 1 canned tuna (3 in total)
Scenario 2	3 Fresh fish + 1 canned tuna (4 in total)
Scenario 3	2 Fresh fish + 2 canned tuna (4 in total)

**Table 2:** Differences in terms of key nutrients across different scenarios, considering 2500, 2000 and 1600 kcal as energy thresholds. Data are divided between MED_EAT-IT and FBDG-based scenarios.

Parameter	Dietary Pattern (kcal)	Baseline (3 Fresh + 0 Can)	Scenario 1 (2 Fresh + 1 Can)	Scenario 2 (3 Fresh + 1 Can)	Scenario 3 (2 Fresh + 2 Can)
**Cholesterol (mg)**	MED 2500	276.6	267.3	265.9	256.5
	MED 2000	269.7	260.4	259.0	249.7
	MED 1600	202.0	192.6	191.3	182.0
	FBDG 2000	220.4	206.7	208.4	194.7
**EPA + DHA (mg)**	MED 2500	697.5	562.6	798.5	663.6
	MED 2000	697.5	562.6	798.5	663.6
	MED 1600	584.2	453.8	666.9	532.0
	FBDG 2000	668.7	470.8	643.8	445.9
**Vit. B12 (μg)**	MED 2500	7.4	6.3	8.2	7.1
	MED 2000	7.5	6.4	8.3	7.2
	MED 1600	6.5	5.3	7.0	6.0
	FBDG 2000	7.2	5.7	7.1	5.5
**Vit. D (μg)**	MED 2500	4.1	3.6	4.6	4.1
	MED 2000	4.0	3.4	4.5	3.9
	MED 1600	3.6	2.8	3.7	3.2
	FBDG 2000	4.1	3.3	4.0	3.2
**Sodium (mg)**	MED 2500	1603	1587	1602	1586
	MED 2000	1312	1296	1311	1295
	MED 1600	1114	1100	1113	1098
	FBDG 2000	1821	1798	1811	1788
**Calcium (mg)**	MED 2500	1057	1050	1063	1056
	MED 2000	1003	996	1009	1001
	MED 1600	1002	996	1007	1000
	FBDG 2000	1273	1263	1272	1261
**Iron (mg)**	MED 2500	18.9	18.6	19.0	18.7
	MED 2000	15.9	15.6	16.0	15.7
	MED 1600	12.7	12.4	12.8	12.5
	FBDG 2000	17.4	17.0	17.3	16.9
**Zinc (mg)**	MED 2500	12.5	12.2	12.2	12.0
	MED 2000	11.0	10.7	10.8	10.5
	MED 1600	9.5	9.3	9.3	9.1
	FBDG 2000	11.8	11.4	11.5	11.1

Note. MED 2500: MED_EAT-IT based scenario with 2500 as energy threshold; MED 2000: MED_EAT-IT based scenario with 2000 as energy threshold; MED 1600: MED_EAT-IT based scenario with 1600 as energy threshold; FBDG 2000: FBDG-based scenario with 2000 as energy threshold; Fresh: portion of fresh fish; Can: portion of canned fish (i.e., canned tuna).

## Data Availability

The original contributions presented in this study are included in the article/Supplementary Material. Further inquiries can be directed to the corresponding author(s).

## Supplementary Materials

The following supporting information can be downloaded at https://www.mdpi.com/article/10.3390/nu18193182/s1. Table S1: Dietary Reference Values (DRVs) considered for this study; Table S2: Frequency of consumption and portion size of MED_EAT-IT dietary pattern, based on an intake of 2500, 2000, 1600 kcal/die; Table S3: Frequency of consumption and portion size of FBDGs-based dietary pattern, based on an intake of 2000 kcal/die; Table S4: Nutritional profile of MED_EAT-IT based scenarios based on an intake of 2500 kcal/die; Table S5: Nutritional profile of MED_EAT-IT based scenarios based on an intake of 2000 kcal/die; Table S6: Nutritional profile of MED_EAT-IT based scenarios based on an intake of 1600 kcal/die; Table S7: Nutritional profile of Italian FBDGs based scenarios based on an intake of 2000 kcal/die; Table S8: Environmental impact of MED_EAT-IT (considering all the three energy targets) and FBDGs based scenarios; Table S9: Absolute and relative differences in Carbon Footprint (CF) and Water Footprint (WF), considering median values, between each scenario and its respective baseline, by dietary pattern and energy target; Table S10: Canned fish consumption in Italy and other European countries and Table S11: Fresh seafood consumption in Italy [28,47].
