# Supplementary material for: Fish Consumption and Sustainability: A Modeling Study Evaluating the Effects of Fish and Canned Fish Consumption on Nutritional Adequacy and Selected Metrics of Environmental Impact
Source: Nutrients. 2026 Sep 26;18(19):3182. doi: 10.3390/nu18193182 (PMC13666764; doi:10.3390/nu18193182)
Supplement: Supplementary file 1 [file nutrients-18-03182-s001.zip › nutrients-4533706-Tables S1-S9.pdf]

Supplementary S1. Dietary Reference Values (DRVs) considered for this study

Nutrient	Target population	Dietary reference value	Reference type
**EPA + DHA**	Adults 18-64 years	250 mg/day	AI
**Vitamin B12**	Adults 18-64 years	4.0 µg/day	AI
**Vitamin D**	Adults 18-64 years	15 µg/day	PRI
**Sodium**	Adults 18-64 years	2.0 g/day	SDT
**Calcium**	Adults 18-64 years	950 mg/day	PRI
**Iron**	Adult males 18-64 years	10 mg/day	PRI
**Iron**	Adult females 18-64 years (pre-menopause)	18 mg/day	PRI
**Zinc**	Adult males 18-64 years	12 mg/day	PRI
**Zinc**	Adults females 18-64 years	9 mg/day	PRI

DRVs were retrieved from the Italian Society of Human Nutrition (SINU) reference values (LARN) (SINU LARN - Livelli Di Assunzione Di Rifeirimento Di Nutrienti Ed Energia; V.; Biomedia: Milano, 2024; ISBN 978-88-86154-76-5). AI: Adequate Intake; PRI: Population Reference Intake; SDT: Suggested Dietary Target.

Supplementary S2. Frequency of consumption and portion size of MED_EAT-IT dietary pattern, based on an intake of 2500, 2000, 1600 kcal/die.

Food category	Food group	2500 kcal		2000 kcal		1600 kcal	
		Frequency of consumption (portions per week)	Portion size (g)	Frequency of consumption (portions per week)	Portion size (g)	Frequency of consumption (portions per week)	Portion size (g)
**Cereals**	Bread	4	194	4	126	4	78
	Pasta	4	129	4	89	4	87
	Rice	2	130	2	90	2	70
	Other cereals (barley, polenta)	2	130	2	90	2	70
	Crispbread (melba toast, rusks)	3.5	52	3.5	43	3.5	33
	Breakfast cereals	3.5	45	3.5	40	3.5	35
**Tubers**	Potatoes	2	199	2	199	1	182
**Red Meat**	Beef	*1/month	97	*1/month	97	*1/month	97
	Pork	*2/month	98	*2/month	98	*2/month	98
	Cured Meat	*1/month	79	*1/month	79	*1/month	70
**Chicken and other poultry**	Chicken and other poultry	2	167	2	167	2	148
**Eggs**	Eggs	2	112	2	112	2	52
**Seafood**	Fish	3	142	3	142	2	121
	Canned fish	0	0	0	0	0	0
**Legumes**	Fresh legumes	4	200	4	181	4	86
**Added fats**	Butter	3	12	3	10	3	8
	Extra virgin olive oil	11	25	11	17	11	10
**Vegetables**	Vegetables	14	186	14	161	14	162
**Fruits**	Fruits	14	179	14	145	14	125
**Dairy**	Milk	3.5	200	4	217	4	217
	Yogurt	3.5	201	4	219	4	219

	Fresh Cheeses	1	124	1	124	1	251
	Semi-hard and hard cheeses	1	75	1	75	1	75
**Nuts**	Nuts	7	30	7	25	7	20
**Sugars**	Biscuits	3	27	3	20	3	17
	Brioche, cakes and bakery product	2	50	1	50	1	25
	Fruit juices	1	183	2	203	1	197

Data reported in the table are the results of the mathematical modeling applied and were not adjusted to fit standardized portion size as indicated by dietary guidelines. *red meat (mammals' meat) is included in the MED_EAT-IT dietary pattern only once per week, limiting in particular beef and cured meat to a max of 1 per month.

Supplementary S3. Frequency of consumption and portion size of FBDGs-based dietary pattern, based on an intake of 2000 kcal/die.

Food category	Food group	Frequency of consumption (g/week)	Portion size (g) – 2000 kcal
**Cereals**	Bread	7	175
	Pasta	4	120
	Rice	2	120
	Other cereals (barley, polenta)	1	120
	Crispbread (melba toast, rusks) *	1	30
	Breakfast cereals *	2	30
**Tubers**	Potatoes	2	200
**Red meat**	Red meat	1	100
**Chicken and other poultry**	Chicken and other poultry	2	100
**Eggs**	Eggs	2	75
**Seafood**	Fish	2	150
	Canned fish *	1	50
**Legumes**	Fresh legumes	3	150
**Added fats**	Butter *	2	15
	Extra virgin olive oil	12	15
**Vegetables**	Vegetables	14	250
**Fruits**	Fruits	14	225
**Dairy**	Milk	3.5	375
	Yogurt	3.5	375
	Fresh Cheeses	2	70
	Semi-hard and hard cheeses	1	50
**Nuts**	Nuts	2	30
**Sugars**	Biscuits	1	30
	Brioche, cakes and bakery product	1	50
	Fruit juices	0	0

*the FBDGs allow flexibility within this food category, whereby a lower consumption frequency of one food group may be compensated for by a proportionally higher consumption of the other food groups included in the same food category.

Supplementary S4. Nutritional profile of MED_EAT-IT based scenarios based on an intake of 2500 kcal/die.

Parameter	MED_EAT-IT Baseline (3 Fresh fish + 0 canned tuna)	MED_EAT-IT Scenario 1 (2 Fresh fish + 1 canned tuna)	MED_EAT-IT Scenario 2 (3 Fresh fish + 1 canned tuna)	MED_EAT-IT Scenario 3 (2 Fresh fish + 2 canned tuna)
% energy from protein	15.1	14.8	14.8	14.4
% energy from lipids	33.6	33.4	33.4	33.1
% energy from carbohydrates (including energy from fibers)	51.2	51.8	51.9	52.4
% energy from total sugars	10.7	10.6	10.7	10.7
Total fibers (g)	41.4	41.4	41.4	41.4
Total fibers/1000 kcal	16.5	16.5	16.5	16.5
Cholesterol (mg)	276.6	267.3	265.9	256.5
% energy from SFA	8.0	7.9	7.9	7.8
% energy from MUFA	16.9	16.8	16.8	16.7
% energy from PUFA	5.2	5.1	5.1	5.1
% energy from ω-6	4.3	4.3	4.2	4.2
% energy from ω-3	0.8	0.8	0.9	0.8
EPA + DHA (mg)	697.5	562.6	798.5	663.6
Total water (ml)	3114	3109	3113	3107
Sodium (mg)	1603	1587	1602	1586
Potassium (mg)	4250	4207	4225	4182
Calcium (mg)	1057	1050	1063	1056
Phosphorus (mg)	1792	1742	1803	1754
Magnesium (mg)	438.7	435.3	436.7	433.4
Iron (mg)	18.9	18.6	19.0	18.7
Estimated heam iron (mg)	1.1	1.0	1.0	0.9
Zinc (mg)	12.5	12.2	12.2	12.0

**Iodine (µg)**	163.0	155.5	169.6	162.1
**Vit. A (retinol eq.) (mg)**	1257	1249	1265	1257
**Vit. D (µg)**	4.1	3.6	4.6	4.1
**Vit. E (tocopherols, tocotrienols) (mg)**	18.6	18.5	18.7	18.6
**Vitamin K (µg)**	57.7	57.6	57.7	57.7
**Vit. B1 (thiamine) (mg)**	1.9	1.9	1.9	1.8
**Vit. B2 (riboflavin) (mg)**	1.9	1.9	1.9	1.9
**Vit. B3 (niacin) (mg)**	23.2	22.7	22.4	21.9
**Vit. B6 (pyridoxine) (mg)**	2.5	2.4	2.4	2.4
**Vit. B9 (folic acid) (mg)**	548.7	547.2	546.7	545.2
**Vit. B12 (µg)**	7.4	6.3	8.2	7.1
**Vit. C (ascorbic acid) (mg)**	262.9	262.7	263.1	262.9

Supplementary S5. Nutritional profile of MED_EAT-IT based scenarios based on an intake of 2000 kcal/die.

Parameter	MED_EAT-IT Baseline (3 Fresh fish + 0 canned tuna)	MED_EAT-IT Scenario 1 (2 Fresh fish + 1 canned tuna)	MED_EAT-IT Scenario 2 (3 Fresh fish + 1 canned tuna)	MED_EAT-IT Scenario 3 (2 Fresh fish + 2 canned tuna)
% energy from protein	16.7	16.2	16.2	15.8
% energy from lipids	33.5	33.3	33.2	32.9
% energy from carbohydrates (including energy from fibers)	49.8	50.5	50.6	51.3
% energy from total sugars	12.2	12.2	12.3	12.2
Total fibers (g)	33.6	33.6	33.6	33.6
Total fibers/1000 kcal	16.8	16.8	16.8	16.8
Cholesterol (mg)	269.7	260.4	259.0	249.7
% energy from SFA	8.6	8.5	8.5	8.4
% energy from MUFA	15.9	15.8	15.8	15.7
% energy from PUFA	5.3	5.2	5.2	5.1
% energy from ω-6	4.3	4.3	4.2	4.1
% energy from ω-3	0.9	0.8	0.9	0.9
EPA + DHA (mg)	697.5	562.6	798.5	663.6
Total water (ml)	2663	2657	2661	2656
Sodium (mg)	1312	1296	1311	1295
Potassium (mg)	3797	3754	3772	3729
Calcium (mg)	1003	996	1009	1001
Phosphorus (mg)	1604	1555	1615	1566
Magnesium (mg)	359.0	355.7	357.0	353.7
Iron (mg)	15.9	15.6	16.0	15.7
Estimated heam iron (mg)	1.1	1.0	1.0	0.9
Zinc (mg)	11.0	10.7	10.8	10.5
Iodine (μg)	176.0	168.5	182.6	175.1

**Vit. A (retinol eq.) (mg)**	1147	1139	1156	1147
**Vit. D (μg)**	4.0	3.4	4.5	3.9
**Vit. E (tocopherols, tocotrienols) (mg)**	14.4	14.3	14.5	14.4
**Vitamin K (μg)**	43.0	43.0	43.0	43.0
**Vit. B1 (thiamine) (mg)**	1.6	1.6	1.6	1.6
**Vit. B2 (riboflavin) (mg)**	1.8	1.8	1.8	1.7
**Vit. B3 (niacin) (mg)**	19.6	19.1	18.8	18.3
**Vit. B6 (pyridoxine) (mg)**	2.2	2.2	2.2	2.1
**Vit. B9 (folic acid) (mg)**	477.8	476.3	475.8	474.3
**Vit. B12 (μg)**	7.5	6.4	8.3	7.2
**Vit. C (ascorbic acid) (mg)**	235.5	235.4	235.7	235.6

Supplementary S6. Nutritional profile of MED_EAT-IT based scenarios based on an intake of 1600 kcal/die.

Parameter	MED_EAT-IT Baseline (3 Fresh fish + 0 canned tuna)	MED_EAT-IT Scenario 1 (2 Fresh fish + 1 canned tuna)	MED_EAT-IT Scenario 2 (3 Fresh fish + 1 canned tuna)	MED_EAT-IT Scenario 3 (2 Fresh fish + 2 canned tuna)
**% energy from protein**	17.9	17.3	17.3	16.7
**% energy from lipids**	33.4	33.1	33.0	32.7
**% energy from carbohydrates (including energy from fibers)**	48.7	49.6	49.7	50.6
**% energy from total sugars**	13.0	13.0	13.1	13.0
**Total fibers (g)**	25.8	25.8	25.8	25.8
**Total fibers/1000 kcal**	16.2	16.2	16.2	16.2
**Cholesterol (mg)**	202.0	192.6	191.3	182.0
**% energy from SFA**	9.9	9.8	9.7	9.6
**% energy from MUFA**	14.3	14.2	14.2	14.1
**% energy from PUFA**	5.2	5.1	5.1	5.0
**% energy from ω-6**	4.1	4.1	4.0	4.0
**% energy from ω-3**	1.0	0.9	1.0	0.9
**EPA + DHA (mg)**	584.2	453.8	666.9	532.0
**Total water (ml)**	2714	2708	2711	2706
**Sodium (mg)**	1114	1100	1113	1098
**Potassium (mg)**	3148	3096	3113	3070
**Calcium (mg)**	1002	996	1007	1000
**Phosphorus (mg)**	1382	1335	1390	1341
**Magnesium (mg)**	320.5	317.1	318.3	315.0
**Iron (mg)**	12.7	12.4	12.8	12.5
**Estimated heam iron (mg)**	0.9	0.8	0.9	0.8
**Zinc (mg)**	9.5	9.3	9.3	9.1
**Iodine (μg)**	157.6	151.2	164.0	156.4

**Vit. A (retinol eq.) (mg)**	1048	1041	1057	1049
**Vit. D (µg)**	3.6	2.8	3.7	3.2
**Vit. E (tocopherols, tocotrienols) (mg)**	10.6	10.5	10.6	10.5
**Vitamin K (µg)**	31.7	31.7	31.7	31.7
**Vit. B1 (thiamine) (mg)**	1.2	1.2	1.2	1.2
**Vit. B2 (riboflavin) (mg)**	1.6	1.6	1.6	1.6
**Vit. B3 (niacin) (mg)**	16.1	15.6	15.4	14.8
**Vit. B6 (pyridoxine) (mg)**	1.8	1.8	1.8	1.7
**Vit. B9 (folic acid) (mg)**	389.4	387.8	387.3	385.8
**Vit. B12 (µg)**	6.5	5.3	7.0	6.0
**Vit. C (ascorbic acid) (mg)**	197.9	197.8	198.1	198.0

Supplementary S7. Nutritional profile of Italian FBDGs based scenarios based on an intake of 2000 kcal/die.

Parameter	FBDGs Baseline (3 Fresh fish + 0 canned tuna)	FBDGs Scenario 1 (2 Fresh fish + 1 canned tuna)	FBDGs Scenario 2 (3 Fresh fish + 1 canned tuna)	FBDGs Scenario 3 (2 Fresh fish + 2 canned tuna)
% energy from protein	16.4	15.9	15.9	15.4
% energy from lipids	25.9	25.6	25.6	25.3
% energy from carbohydrates (including energy from fibers)	57.7	58.6	58.5	59.3
% energy from total sugars	14.2	14.2	14.2	14.2
Total fibers (g)	40.2	40.2	40.2	40.2
Total fibers/1000 kcal	18.7	18.7	18.7	18.8
Cholesterol (mg)	220.4	206.7	208.4	194.7
% energy from SFA	7.4	7.4	7.3	7.3
% energy from MUFA	12.3	12.2	12.2	12.1
% energy from PUFA	3.5	3.4	3.4	3.3
% energy from ω-6	2.6	2.6	2.6	2.6
% energy from ω-3	0.8	0.7	0.8	0.7
EPA + DHA (mg)	668.7	470.8	643.8	445.9
Total water (ml)	3103	3093	3098	3088
Sodium (mg)	1821	1798	1811	1788
Potassium (mg)	4572	4509	4531	4467
Calcium (mg)	1273	1263	1272	1261
Phosphorus (mg)	1720	1648	1696	1624
Magnesium (mg)	387.2	382.3	384.0	379.1
Iron (mg)	17.4	17.0	17.3	16.9
Estimated heam iron (mg)	0.9	0.8	0.8	0.7
Zinc (mg)	11.8	11.4	11.5	11.1
Iodine (μg)	227.4	216.3	226.5	215.5

**Vit. A (retinol eq.) (mg)**	1472	1460	1472	1460
**Vit. D (μg)**	4.1	3.3	4.0	3.2
**Vit. E (tocopherols, tocotrienols) (mg)**	13.5	13.4	13.5	13.4
**Vitamin K (μg)**	53.2	53.2	53.2	53.2
**Vit. B1 (thiamine) (mg)**	1.6	1.5	1.5	1.5
**Vit. B2 (riboflavin) (mg)**	2.2	2.2	2.2	2.1
**Vit. B3 (niacin) (mg)**	21.7	21.0	20.9	20.2
**Vit. B6 (pyridoxine) (mg)**	2.5	2.4	2.4	2.4
**Vit. B9 (folic acid) (mg)**	589.0	586.9	587.0	584.8
**Vit. B12 (μg)**	7.2	5.7	7.1	5.5
**Vit. C (ascorbic acid) (mg)**	313.1	312.8	313.1	312.8

Supplementary S8. Environmental impact of MED_EAT-IT (considering all the three energy targets) and FBDGs based scenarios.

Scenario (kcal)	CF Median (Q1 – Q3)	WF Median (Q1 – Q3)	*WF Median (Q1 – Q3)
**MED_EAT_IT Baseline (2500kcal)**	2.82 (2.11 – 3.77)	3325 (2808 – 4244)	3128 (2628 – 3987)
**MED_EAT_IT Scenario 1 (2500kcal)**	2.74 (2.06 – 3.63)	3281 (2768 – 4187)	3128 (2628 – 3987)
**MED_EAT_IT Scenario 2 (2500kcal)**	2.82 (2.09 – 3.82)	3233 (2712 - 4167)	2989 (2489 – 3848)
**MED_EAT_IT Scenario 3 (2500kcal)**	2.73 (2.04 – 3.68)	3189 (2672 – 4109)	2989 (2489 – 3848)
**MED_EAT_IT Baseline (2000kcal)**	2.67 (1.99 – 3.57)	2949 (2493 - 3739)	2753 (2313 – 3482)
**MED_EAT_IT Scenario 1 (2000kcal)**	2.59 (1.95 – 3.43)	2905 (2452 – 3681)	2753 (2313 – 3482)
**MED_EAT_IT Scenario 2 (2000kcal)**	2.67 (1.97 – 3.62)	2857 (2396 – 3662)	2614 (2174 – 3343)
**MED_EAT_IT Scenario 3 (2000kcal)**	2.59 (1.93 – 3.48)	2813 (2356 – 3604)	2614 (2174 – 3343)
**MED_EAT_IT Baseline (1600kcal)**	2.35 (1.79 – 3.11)	2453 (2073 – 3153)	2284 (1918 – 2930)
**MED_EAT_IT Scenario 1 (1600kcal)**	2.26 (1.73 – 2.95)	2404 (2028 – 3088)	2284 (1918 – 2930)
**MED_EAT_IT Scenario 2 (1600kcal)**	2.33 (1.75 – 3.13)	2361 (1978 – 3070)	2158 (1792 – 2805)
**MED_EAT_IT Scenario 3 (1600kcal)**	2.25 (1.71 – 2.99)	2317 (1937 – 3012)	2158 (1792 – 2805)
**FBDGs Baseline (2000kcal)**	2.91 (2.19 – 3.92)	3021 (2567 – 4016)	2825 (2387 – 3759)
**FBDGs Scenario 1 (2000kcal)**	2.78 (2.11 – 3.70)	2957 (2508 – 3931)	2825 (2387 – 3759)
**FBDGs Scenario 2 (2000kcal)**	2.85 (2.14 – 3.86)	2940 (2487 – 3935)	2744 (2307 – 3678)
**FBDGs Scenario 3 (2000kcal)**	2.73 (2.07 – 3.65)	2876 (2428 – 3851)	2744 (2307 – 3678)

* values of WF considering wild-caught fish and seafood products instead of aquaculture farming, assuming a water footprint of zero (Gephart, J.A.; Pace, M.L.; D'Odorico, P. Freshwater savings from marine protein consumption. Environ. Res. Lett. 2014, 9, 014005. https://doi.org/10.1088/1748-9326/9/1/014005)

Supplementary S9. Absolute and relative differences in Carbon Footprint (CF) and Water Footprint (WF), considering median values, between each scenario and its respective baseline, by dietary pattern and energy target.

Dietary pattern (kcal)	Scenario	ΔCF (kg CO_2 eq./day)	ΔCF (%)	ΔWF (L/day)	ΔWF (%)	ΔWF* (L/day)	ΔWF* (%)
**MED_EAT-IT (2500)**	Scenario 1	−0.08	−2.8%	−44	−1.3%	0	0.0%
**MED_EAT-IT (2500)**	Scenario 2	0.00	0.0%	−92	−2.8%	-139	-4.4%
**MED_EAT-IT (2500)**	Scenario 3	−0.09	−3.2%	−136	−4.1%	-139	-4.4%
**MED_EAT-IT (2000)**	Scenario 1	−0.08	−3.0%	−44	−1.5%	0	0.0%
**MED_EAT-IT (2000)**	Scenario 2	0.00	0.0%	−92	−3.1%	-139	-5.0%
**MED_EAT-IT (2000)**	Scenario 3	−0.08	−3.0%	−136	−4.6%	-139	-5.0%
**MED_EAT-IT (1600)**	Scenario 1	−0.09	−3.8%	−49	−2.0%	0	0.0%
**MED_EAT-IT (1600)**	Scenario 2	−0.02	−0.9%	−92	−3.7%	-126	-5-5%
**MED_EAT-IT (1600)**	Scenario 3	−0.10	−4.3%	−136	−5.5%	-126	-5-5%
**FBDGs (2000)**	Scenario 1	−0.13	−4.5%	−64	−2.1%	0	0.0%
**FBDGs (2000)**	Scenario 2	−0.06	−2.1%	−81	−2.7%	-81	-2.9%
**FBDGs (2000)**	Scenario 3	−0.18	−6.2%	−145	−4.8%	-81	-2.9%

* values of WF considering wild-caught fish and seafood products instead of aquaculture farming, assuming a water footprint of zero (Gephart, J.A.; Pace, M.L.; D'Odorico, P. Freshwater savings from marine protein consumption. Environ. Res. Lett. 2014, 9, 014005. https://doi.org/10.1088/1748-9326/9/1/014005)
